# Effects of a small-sided games training program in youth male soccer players: variations of the locomotor profile while interacting with baseline level and with the accumulated load

**DOI:** 10.1186/s13102-022-00595-y

**Published:** 2022-11-23

**Authors:** Ana Filipa Silva, Rafael Oliveira, Halil Ibrahim Ceylan, Zeki Akyildiz, Francisco Tomás González-Fernández, Hadi Nobari, Mehmet Yıldız, Sabri Birlik, Filipe Manuel Clemente

**Affiliations:** 1 Instituto Politécnico de Viana do Castelo, Rua Escola Industrial e Comercial de Nun’Álvares, Escola Superior Desporto e Lazer, 4900-347 Viana do Castelo, Portugal; 2 Research Center in Sports Performance, Recreation, Innovation and Technology (SPRINT), 4960-320 Melgaço, Portugal; 3 The Research Centre in Sports Sciences, Health Sciences and Human Development (CIDESD), 5001-801 Vila Real, Portugal; 4 Sports Science School of Rio Maior–Polytechnic Institute of Santarém, 2040-413 Rio Maior, Portugal; 5 Life Quality Research Centre, 2040-413 Rio Maior, Portugal; 6 Physical Education and Sports Teaching Department, Kazim Karabekir Faculty of Education, Ataturk University, Erzurum, Turkey; 7 Sports Science Department, Gazi University, Ankara, Turkey; 8 Department of Physical Education and Sport, Faculty of Education and Sport Sciences, University of Granada, Campus of Melilla, 52006 Melilla, Spain; 9 Department of Exercise Physiology, Faculty of Educational Sciences and Psychology, University of Mohaghegh Ardabili, Ardabil, 5619911367 Iran; 10 Faculty of Sport Sciences, University of Extremadura, 10003 Cáceres, Spain; 11 Department of Motor Performance, Faculty of Physical Education and Mountain Sports, Transilvania University of Braşov, 500068 Brasov, Romania; 12 Afyon Kocatepe University Sports Science Faculty, Afyon, Turkey; 13 Instituto de Telecomunicações, Delegação da Covilhã, 1049-001 Lisbon, Portugal

**Keywords:** Football, Drill-based games, Heart rate, Global positioning systems, Athletic performance, Physical fitness

## Abstract

**Purpose:**

This study aimed to (1) analyze the impact of a small-sided game training program in the locomotor profile of youth male soccer players (while interacting with the baseline level – higher and lower level); and (2) test the relationships between variation in locomotor profile and the accumulated demands in 3v3, 5v5 and match over the period of observation.

**Methods:**

The cohort lasted 3-weeks. Twenty under-17 male amateur soccer players (16.8 ± 0.41 years; experience: 6.35 ± 0.67 years) were assessed twice for their final velocity at 30−15 intermittent fitness test (VIFT), peak speed at 30-m sprint test (PSS) and anaerobic speed reserve (ASR). The PSS was estimated using a Global Positioning System, while the VIFT was estimated using the maximum level attained by the players during the test. Based on the baseline levels, the scores were standardized using the Z-score. The total score of athleticism (TSA) was calculated per player to organize the players into two groups: lower TSA and higher TSA. Over the three weeks of observation, the small-sided games of 3v3 and 5v5 and match demands were monitored using polar team pro. The heart rate responses (mean and peak), distance covered (overall and split by speed thresholds), and peak speed in these games were obtained and summed over the weeks. The repeated measures ANCOVA tested the variations (time) of the locomotor profile of players while considering the baseline as covariable and the group as a factor. The Pearson-product correlation test analyzed the relationships between variations in locomotor profile (Δ, post-baseline) and the accumulated demands in 3v3, 5v5, and match.

**Results:**

Between-groups analysis (lower TSA vs. higher TSA) revealed no significant differences on VIFT (*p* = 0.915), PSS (*p* = 0.269), ASR (*p* = 0.258) and TSA score (*p* = 0.138). Within-group (baseline vs. post-observation) analysis revealed significant difference on VIFT (*p* < 0.001), PSS (*p* = 0.008), while no significant differences were found on ASR (*p* = 0.949) and TSA score (*p* = 0.619). Significant correlations were found between ΔPSS and match total distance (r = 0.444; *p* = 0.050), match Z2 (r = 0.481; *p* = 0.032) and match Z3 (r = 0.454; *p* = 0.044). Significant correlations were found between ΔTSA and match total distance (r = 0.457; *p* = 0.043), match Z1 (r = 0.451; *p* = 0.046), match Z2 (r = 0.500; p = 0.025) and match Z3 (r = 0.468; *p* = 0.037).

**Conclusion:**

Significant improvements were observed after the period of observation. However, the fitness baseline level and the accumulated training load in the small-sided games seem to have no significant impact on the observed improvements.

**Supplementary Information:**

The online version contains supplementary material available at 10.1186/s13102-022-00595-y.

## Introduction

Soccer is a team sport characterized by predominantly low-intensity and short-term high-intensity activity with a large number of repetitions [1, 2]. Young elite soccer players covered a distance of approximately 6.5–8.7 km during the match, of which about 671–991 m were high-intensity activities, and 186–449 m were sprint distances [3]. Thus, a soccer match can be defined as an intermittent exercise, while periods of low-to-moderate efforts are interspaced by maximal to near-maximal actions [4, 5]. Based on the high-demanding context of soccer, players need to have well-developed aerobic and anaerobic power to be able to sustain 90 min of high-intensity explosive activities such as sprinting, changing directions, accelerations, decelerations, etc., and, in short, to cope with the demands of the game [6–9]. Higher levels of aerobic capacity enhance rapid recovery from explosive high-intensity intermittent movements through the increased aerobic response, improved lactate removal, and enhanced PCr regeneration and may also be a prerequisite for increasing the efficiency of anaerobic capacity when performing these efforts [10].

In the literature, parameters such as peak sprint speed (PSS) and maximal aerobic speed (MAS; the minimum speed at which maximum oxygen uptake is obtained), which form locomotor profiles in soccer and are associated with aerobic and anaerobic performance characteristics, are frequently used to predict high-intensity exercise profiles (both continuous and intermittent) and repetitive sprint performance of the players [11, 12]. The MAS reflects a player’s maximum aerobic power integrated with running economy. PSS, also called maximal anaerobic speed, indicates the highest running speed achieved with maximum anaerobic energy release [9]. Since the decisive movements in soccer occur in small areas, PSS is essential in locomotor activities that require high power [13, 14]. Moreover, a previous study conducted on young soccer players demonstrated that the most common movement before scoring a goal was sprinting [15]. Another essential quality for a player’s physical performance is the combination of MAS and PSS, typically mentioned as the anaerobic speed reserved (ASR) [16]. The ASR represents the difference between MAS and PSS, another important variable affecting the tolerance to repeated high-intensity exercise [17]. A higher ASR (Lower anaerobic energy contribution) leads to a prolonged exhaustion time, less metabolic and peripheral fatigue, and, as a result, improved sportive performance at exercise intensities above maximal aerobic speed [16, 18].

Previous studies showed that aerobic and anaerobic performance parameters (ventilatory anaerobic threshold, heart rate, lactate, VO2max and running velocity at VO2max) increased after the pre-season period due to high aerobic-type training volume and did not change during the competitive period in professional soccer players [8, 19], and in youth soccer players [20–22]. Regarding the anaerobic power, it was observed that it increased from the beginning of the season to the middle of the season and remained unchanged from the middle of the season to the end of the season in semi-professional soccer players [23]. In addition, some studies found that sprint performance decreased in the middle of the season compared to the beginning of the season [23]. In contrast, others observed that sprint performance increased in the middle and end of the season compared to the start of the season [20, 24]. The differences can explain the reason for the inconsistent results mentioned above among the participants, the differences in the volume and intensity of the training, and the focus of the trainers to develop specific conditioning components during particular periods of the season [19].

The development of the physical fitness characteristics of the players mentioned above throughout the season requires an optimal dose-response relationship. The dose-response relationship may differ depending on the players’ trainability, the training stimuli’s suitability, the season’s stage, and even how well the exercises are tailored to each player [25]. Recent studies showed that the accumulated training load plays a critical role in physical fitness changes throughout the season in professional soccer players [26, 27] and young soccer players [28]. Previous studies found positive correlations between the perceived effort or accumulated training load and the improvements in the velocity achieved at 30−15 Intermittent Fitness Test [29], and MAS capacity determined by using the 5-min test in professional soccer players [30]. Trainers often use small-sided games (SSGs) to improve players’ capacity for high-intensity interval training [31, 32]. Some studies examined the relationships between aerobic fitness tests and external and internal training load measures during SSGs. For instance; Owen et al. [32] indicated that a positive correlation was found between aerobic capacity performance measured by the YoYoIR1 test and the total distance covered, high metabolic power distance (m; ≥ 20 W kg^−1^) covered during small-sided games (using 5 vs. 5 formats) in professional soccer players. Another study conducted by Younesi et al. [33] reported that there were relationships between VIFT and external loads, such as total distance covered, high-intensity running, and mechanical work during SSGs using the 3 vs. 3 format.

Responses to training doses throughout the season (the dose-response relationship) may vary depending on players’ initial physical fitness characteristics, and these responses can vary widely among players; this is often described as “high and low responders” [33, 34]. In this context, analysis of responding or non-responding players, i.e., determining the player’s responsiveness and unresponsiveness to training load, can help explain physical fitness changes [35]. Clemente et al. [30] reported that baseline levels of soccer players influenced variations in physical fitness throughout the season. These variations were largely related to the sessions’ perceived effort and training load. Subsequently, the same researchers indicated that low responders experienced a decrease in MAS capacity throughout the season, while high responders observed gradual improvements in MAS capacity (~ 21%) throughout the season. As far as we know, no study evaluates the variations of variables such as MAS, PSS, and ASR in young soccer players according to TSA (total score of athleticism) and correlates the variations in these variables with the accumulated external loads during small-sided games (SSGs; e.g., 3v3, 5v5) and matches. The potential relationships between these variables may assist coaches in using SSGs as a more ecological and time-efficient monitoring tool for players’ physiological profile and performance capacity [36–38]. They may also serve as a complement to traditional running-based assessments [33, 37]. However, understanding the impact of SSGs on locomotor profile variations requires a monitoring process to inspect a dose-response relationship potentially. Trying to understand which factor can play a more critical role in interpreting locomotor profile, this study tested the impact of physical fitness level (at baseline level) and the demands occurring in the small-sided games and matches. This can offer an opportunity to identify the real impact of each factor on the adaptations occurring in the locomotor profile. Thus, the aim of this study was two-fold: (This study aimed to (i) analyze the impact of a small-sided game training program in the locomotor profile of youth male soccer players (while interacting with the baseline level – higher and lower level); and (ii) test the relationships between variation in locomotor profile and the accumulated demands in 3v3, 5v5 and match over the period of observation.

## Materials and methods

### Study design

This study followed a prospective cohort design. Players were selected by convenience sampling. The study protocol was approved by the Institutional Review Board of Afyon Kocatepe University (protocol code AKU-2021/2) and followed the Declaration of Helsinki ethical standards for the study in humans. Since the study included minors, the parents signed the consent form, and the study information was previously provided to both players and the parents.

### Setting and procedures

**Fig. 1:**
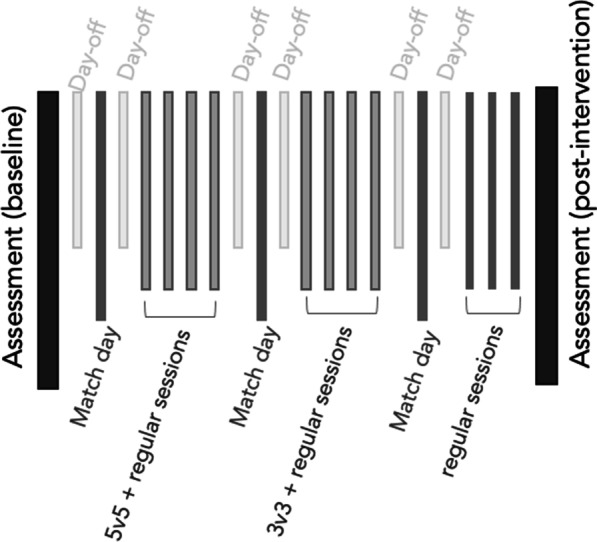
Study timeline

The study lasted three weeks (Fig. 1). The study occurred in the mid of the competitive season. The players were assessed twice for the final velocity achieved at the 30−15 Intermittent Fitness Test (VIFT) and peak sprint speed (PSS). Between the assessments, players were monitored for their physiological (heart rate responses, HR) and locomotor demands in training sessions and matches. A 24-h rest preceded the assessments. The assessments (baseline and post-observation) started at 5 p.m., with environmental conditions of 10 and 16 °C and 67 and 62% relative humidity, respectively. The assessments were preceded by a standardized warm-up protocol (FIFA 11+) [39]. After that, players immediately performed two trials of the 30-m linear sprint test (interspaced by 5 min rest) and, after 3 min of rest, performed the 30−15 Intermittent Fitness Test.

Regarding the training sessions and matches, they were monitored using the Polar Team Pro. Specifically, during the observation period, the observations focused on the 5v5 and 3v3 small-sided game formats occurring in the first two weeks. The formats were observed since they represented the most common formats selected for working aerobic power (3v3) and endurance workouts (5v5). Moreover, they were also chosen because they were part of the regular training program of the participants. The 3v3 format was performed two times (3 min each) per training session, interspaced by 3 min of rest. The 3v3 was employed in a 39 × 24 m and 32 × 19 m synthetic turf pitch, using a small goal (2 × 1 m) centered in the endline. The 5v5 was also employed two times per session (5 min each), interspaced by 3 min of rest. The 5v5 was played in a 40 × 25 m and 50 × 31 m synthetic turf pitch and a small goal (2 × 1 m) centered in the endline. The players were allocated to the teams based on the coach’s decision, aiming to keep balance in the proficiency level and also trying to have defenders, midfielders, and forwards in each group.

### Participants

Twenty male under-17 amateur soccer players (16.8 ± 0.4 years old; 6.4 ± 0.7 years of experience; 167.9 ± 3.4 centimeters of stature; 65.4 ± 6.4 kg of body mass) voluntarily participated in this study. The eligibility criteria for this study were: (i) players must act as outfield; (ii) players should not present any injury or illness during the observation period; (iii) players must be present in both assessment moments and all training sessions analyzed (5v5 and 3v3 formats of play).

### Independent variable

After the baseline assessment, the best PSS and the VIFT were recorded for each athlete. Additionally, the anaerobic speed reserve (ASR) was calculated using the subtraction of PSS by the VIFT. The scores of each athlete in the three measures (VIFT, PSS, and ASR) were standardized using the z-score, considering the mean and standard deviation of the twenty players. The sum of the z-score of each test allowed us to determine the total score of athleticism (TSA) of each player [40]. Based on the z-scores, the players were classified as: (i) lower TSA if below 0.0; and (ii) higher TSA if above 0.0.

### Peak sprint speed (PSS)

The peak sprint speed (PSS) was measured during a 30-m linear sprint test using the Polar Team Pro, combining a Global Positioning System (GPS) with a heart rate sensor. The Polar Team Pro is accurate and reliable in determining peak speed, as previously revealed in a concurrent-validity study conducted with a radar gun [41]. The PSS obtained in each of the two trials was collected, and the best score was used to further data treatment. The 30-m sprint test was marked on the synthetic turf, and the players always started with the same preferred leg in front. The coefficient of variation presented by the players (within-players variability) in the PSS was 3.0%.

### Final velocity at 30−15 intermittent fitness test (VIFT)

The 30−15 Intermittent Fitness test was employed to determine the ability of players to perform repeated and progressive efforts until exhaustion [42]. The tests consist in to perform repeated and advanced 30-s runs, interspaced by a 15-s rest period. The tests starts at 8 km/h and the pace is increasing at each new 30-s round by 0.5 km/h [43]. The final velocity (VIFT) completed by the player is obtained as the main outcome. The test ends when the player is unable to keep the pace (following the audio beep) by two consecutive trials.

### Anaerobic speed reserve (ASR)

The anaerobic speed reserve (ASR) was calculated by subtracting the PSS by the VIFT [16].

### Within-players variation of PSS, VIFT, ASR, and TSA

The percentage of change (pos-observation – baseline) was calculated for each main outcome using the formula: $\frac{{{\text{post}}\;{\text{observation}} - {\text{baseline}}}}{{{\text{baseline}}}} \times 100$.

### Heart rate (HR) responses and locomotor demands in training and matches

The players were monitored for their demands during the 5v5 games, 3v3 games and match participation. During the period of observation, the players performed 4 training sessions with 5v5 format and another 4 training sessions with 3v3 format (Fig. 1). For each training session, they performed two trials of the format, preceded by the FIFA11 + standardized-warm up. The 5v5 consisted of 5 min per repetition interspaced by 3 rest, while the 3v3 format consisted of 3 min per repetition interspaced by 3 min rest. The 5v5 format was played in 50 × 31 m and 40 × 25 m (each field size was used per each training session with 5v5). The 3v3 format was played in 39 × 24 m and 32 × 19 m (each field size was used per each training session with 3v3 format). Both formats of play did not have regular goals. Small-goals (2 × 1 m) were centered in the end line of each team. The offside rule was not used. The match demands were also monitored during the period of observation. The player’s demands were monitored in all sessions with SSGs and all the matches. The Polar Team Pro was used to monitor each game’s HR mean and HRpeak (1 Hz, Polar, Finland). The mean HRmean and HRpeak were obtained for the 5v5, 3v3, and match. Moreover, the Polar Team Pro was also used to determine the peak speed; distance covered; distance covered at zone 1 (Z1: 3.00–6.99 km/h); distance covered at zone 2 (Z2: 7.00–10.99 km/h); distance covered at zone 3 (Z3: 11.00–14.99 km/h); distance covered at zone 4 (Z4: 15.00–18.99 km/h); and distance covered at zone 5 (Z5: >19.00 km/h). The mean peak speed was calculated for the 5v5, 3v3, and match. The sum of total distance and distances covered at different speed thresholds in all 5v5, 3v3, and match were also calculated for each player.

### Statistical procedures

The mean and standard deviations were used as descriptive statistics. The Shapiro-Wilk and Levene’s test confirmed normality and homogeneity (*p* > 0.05). The repeated measures ANCOVA tested the interaction of time (baseline and post-observation assessments) * group (lower vs. higher TSA) for the variations of VIFT, ASR, PSS, and TSA, while using the baseline level of each measure as covariable. Since the covariable was not significant, a mixed ANOVA (time*group) was tested. The Bonferroni test was used as a post-hoc test. The partial eta squared tested the effect size of repeated measures ANCOVA and mixed ANOVA. In contrast, Cohen’s d test (using pooled standard deviation) was used to analyze the standardized effect size in pairwise comparisons. For the second aim of this study, the mean of HRmean, HRpeak, peak speed, and the sum of total distance and distances covered at different speed thresholds occurring in 5v5, 3v3, and match were correlated with the percentage of variation of VIFT ASR, PSS, and TSA. The correlation was tested using the Pearson-product correlation test. The magnitude of correlation was set as [44]: trivial (0.0–0.1); small (0.1–0.3); moderate (0.3–0.5); large (0.5–0.7); very large (0.7–0.9); and nearly perfect (> 0.9). The statistical procedures were executed in the SPSS software (version 28.0.0.0, IBM, Chicago, USA) for a *p* < 0.05.

## Results

**Fig. 2:**
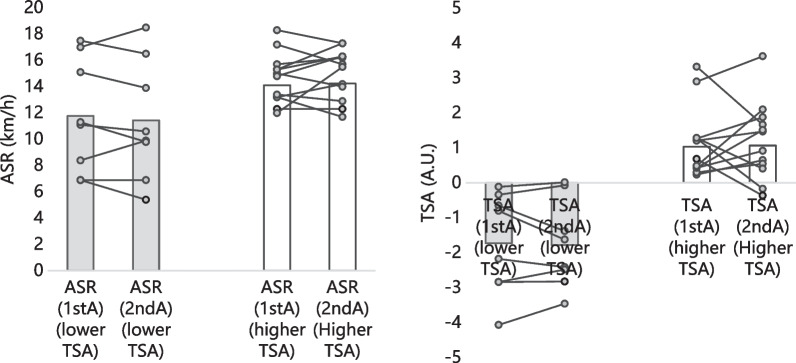
Physical fitness variations of players with lower and higher TSA levels. TSA: total score of athleticism; VIFT: final velocity at 30 − 15 Intermittent Fitness test; ASR: anaerobic speed reserve. (grey bars: group with lower TSA; white bars: group with higher TSA; the lines means the intra-individual variation of each player)

The repeated-measures ANCOVA revealed no significant interaction (factor * baseline level) on VIFT (*F* = 1.584; *p* = 0.225; ${\eta }_{2}^{p}$ = 0.085), PSS (*F *= 0.579; *p* = 0.457; ${\eta }_{2}^{p}$ = 0.033), ASR (*F *= 0.370; *p* = 0.551; ${\eta }_{2}^{p}$ = 0.021), and TSA (*F *= 3.226; *p* = 0.090; ${\eta }_{2}^{p}$ = 0.160). Additionally, mixed ANOVA revealed no significant interaction (factor*TSA group) on VIFT (*F *= 0.012; *p* = 0.915; ${\eta }_{2}^{p}$ = 0.001), PSS (*F *= 1.303; *p* = 0.269; ${\eta }_{2}^{p}$ = 0.071), ASR (*F *= 1.373; *p* = 0.258; ${\eta }_{2}^{p}$ = 0.075), and TSA (*F *= 2.426; *p* = 0.138; ${\eta }_{2}^{p}$ = 0.125). Between-groups analysis (lower TSA vs. higher TSA) revealed no significant differences on VIFT (*p* = 0.915), PSS (*p* = 0.269), ASR (*p* = 0.258) and TSA score (*p* = 0.138). Within-group (baseline vs. post-intervention) analysis revealed significant difference on VIFT (*p *< 0.001), PSS (*p* = 0.008), while no significant differences were found on ASR (*p* = 0.949) and TSA score (*p* = 0.619). The descriptive statistics of both groups in the two moments of assessment can be found in Fig. 2.

The players with lower TSA significantly improved the VIFT (baseline: 14.6 ± 1.6 km/h; post-intervention: 15.7 ± 1.6 km/h; + 7.3%; *p* < 0.001; d = 0.673), and the peak sprint speed (baseline: 26.4 ± 3.0 km/h; post-intervention: 27.1 ± 3.1 km/h; + 2.7%; *p* = 0.428; d = 0.238). The ASR non-significantly decreased in lower TSA group (baseline: 11.8 ± 4.3 km/h; post-intervention: 11.4 ± 4.5 km/h; − 2.9%; *p* = 0.421; d = – 0.076). The players with lower TSA not significantly change the final TSA score (baseline: − 1.7 ± 1.4 A.U.; post-intervention: − 1.8 ± 1.3 A.U.; 3.0%; *p* = 0.180; d = – 0.038).

The players with higher TSA significantly improved the VIFT (baseline: 15.8 ± 1.1 km/h; post-intervention: 16.8 ± 1.0 km/h; + 6.3%; *p* < 0.001; d = 0.991), peak sprint speed (baseline: 29.9 ± 1.5 km/h; post-intervention: 31.0 ± 1.7 km/h; + 3.8%; *p* = 0.006; d = 0.724). The ASR non-significantly increased in higher TSA group (baseline: 14.1 ± 1.9 km/h; post-intervention: 14.2 ± 1.9 km/h; + 0.9%; *p* = 0.363; d = 0.065). The players with higher TSA no-significantly changed the final TSA score (baseline: 1.0 ± 1.0 A.U.; post-intervention: 1.0 ± 1.1 A.U.; 3.1%; *p* = 0.240; d = 0.030).

Pearson-product correlation test explored the relationships between variations in physical fitness levels (post–baseline) and the sum of demands accumulated in 3v3, 5v5, and match contexts (Table 1). Significant correlations were found between ΔPSS and match total distance (r = 0.444 [95%CI: – 0.010; 0.735]; *p* = 0.050), match Z2 (r = 0.481 [95%CI: 0.032; 0.036]; *p* = 0.032) and match Z3 (r = 0.454 [95%CI: 0.003; 0.741]; *p* = 0.044). Significant correlations were found between ΔTSA and match total distance (r = 0.457 [95%CI: 0.007; 0.743]; *p* = 0.043), match Z1 (r = 0.451 [95%CI: – 0.002; 0.739]; *p* = 0.046), match Z2 (r = 0.500 [95%CI: 0.060; 0.766]; *p* = 0.025) and match Z3 (r = 0.468 [95%CI: 0.020; 0.749]; *p* = 0.037). Additional files 1, 2, 3 are available with information on the study.

**Table 1:** Correlation table (r) between Δ of physical fitness and sum of demands occurring in 3v3, 5v5 and match

Variables	ΔVIFT	ΔPSS	ΔASR	ΔTSA
3v3 HRmean	r = 0.182; *p* = 0.443	r = – 0.268; *p* = 0.254	r = – 0.317; *p* = 0.173	r = – 0.214; *p* = 0.366
3v3 HRpeak	r = – 0.017; *p* = 0.943	r = – 0.197; *p* = 0.406	r = – 0.175; *p* = 0.460	r = – 0.188; *p* = 0.427
3v3 TD	r = – 0.240; *p* = 0.309	r = 0.123; *p* = 0.604	r = 0.206; *p* = 0.384	r = 0.059; *p* = 0.806
3v3 peak speed	r = 0.292; *p* = 0.211	r = 0.168; *p* = 0.478	r = 0.044; *p* = 0.855	r = 0.209; *p* = 0.375
3v3 Z1	r = 0.331^#^; *p* = 0.153	r = – 0.191; *p* = 0.420	r = – 0.303^#^; *p* = 0.193	r = – 0.095; *p* = 0.690
3v3 Z2	r = – 0.439^#^; *p* = 0.053	r = 0.159; *p* = 0.503	r = 0.315^#^; *p* = 0.176	r = 0.056; *p* = 0.814
3v3 Z3	r = – 0.198; p = 0.402	r = – 0.170; *p* = 0.473	r = – 0.081; *p* = 0.733	r = – 0.177; *p* = 0.456
3v3 Z4	r = – 0.064; *p* = 0.790	r = 0.060; *p* = 0.803	r = 0.079; *p* = 0.739	r = 0.057; *p* = 0.811
3v3 Z5	r = 0.066; *p* = 0.783	r = 0.243; *p* = 0.301	r = 0.200; *p* = 0.399	r = 0.191; *p *= – 0.207
5v5 HRmean	r = 0.054; *p* = 0.821	r = 0.074; *p* = 0.757	r = 0.047; *p* = 0.843	r = 0.146; *p* = 0.540
5v5 HRpeak	r = 0.122; *p* = 0.609	r = 0.323^#^; *p* = 0.164	r = 0.252; *p* = 0.283	r = 0.399^#^; *p* = 0.081
5v5 TD	r = – 0.364^#^; *p* = 0.114	r = 0.173; *p* = 0.467	r = 0.299; *p* = 0.200	r = 0.128; *p* = 0.592
5v5 peak speed	r = – 0.122; *p* = 0.607	r = – 0.257; *p* = 0.274	r = – 0.191; *p* = 0.420	r = – 0.257; *p* = 0.274
5v5 Z1	r = – 0.092; *p* = 0.698	r = 0.329^#^; *p* = 0.157	r = 0.339^#^; *p* = 0.143	r = 0.188; *p* = 0.427
5v5 Z2	r = – 0.220; *p* = 0.351	r = 0.043; *p* = 0.857	r = 0.124; *p* = 0.602	r = 0.051; *p* = 0.830
5v5 Z3	r = – 0.244; *p* = 0.301	r = 0.119; *p* = 0.617	r = 0.203; *p* = 0.390	r = 0.073; *p* = 0.759
5v5 Z4	r = – 0.340^#^; *p* = 0.143	r = 0.071; *p* = 0.766	r = 0.196; *p* = 0.408	r = 0.017; *p* = 0.945
5v5 Z5	r = 0.001; *p* = 0.998	r = – 0.132; *p* = 0.578	r = – 0.123; *p* = 0.606	r = – 0.006; *p* = 0.980
Match HRmean	r = 0.122; *p* = 0.642	r = 0.051; *p* = 0.845	r = – 0.004; *p* = 0.988	r = 0.120; *p* = 0.646
Match HRpeak	r = 0.022; *p* = 0.933	r = 0.207; *p* = 0.425	r = 0.169; *p* = 0.516	r = 0.305^#^; *p* = 0.234
Match TD	r = 0.227; *p* = 0.335	r = 0.444^#^; *p* = 0.050^*^	r = 0.323; *p* = 0.165	r = 0.457^#^; *p* = 0.043^*^
Match peak speed	r = – 0.068; *p* = 0.795	r = – 0.068; *p* = 0.796	r = – 0.031; *p* = 0.905	r = – 0.080; *p* = 0.760
Match Z1	r = 0.267; *p* = 0.256	r = 0.436^#^; *p* = 0.055	r = 0.301^#^; *p* = 0.197	r = 0.451^#^; *p* = 0.046^*^
Match Z2	r = 0.220; *p* = 0.351	r = 0.481^#^; *p* = 0.032^*^	r = 0.360^#^; *p* = 0.119	r = 0.500^#^; *p* = 0.025^*^
Match Z3	r = 0.175; *p* = 0.459	r = 0.454^#^; *p* = 0.044^*^	r = 0.353^#^; *p* = 0.127	r = 0.468^#^; *p* = 0.037^*^
Match Z4	r = 0.194; *p* = 0.413	r = 0.413^#^; *p* = 0.070	r = 0.307^#^; *p* = 0.187	r = 0.429^#^; *p* = 0.059
Match Z5	r = 0.223; *p* = 0.345	r = 0.243; *p* = 0.303	r = 0.139; *p* = 0.559	r = 0.260; *p* = 0.267

The r-value represents the correlation between measures, and the *p*-value represents the significancy level of the correlation

∆: difference of post-intervention and baseline (post–baseline); *VIFT*, final velocity at 30−15 Intermittent Fitness Test; *PSS*, peak sprint speed; *ASR*, anaerobic speed reserve; *TSA*, total score of athleticism; *HRmean*, mean heart rate; *HRpeak*, peak heart rate; *TD*, total distance; *Z1*, distance covered between 3.00 and 6.99 km/h; *Z2*, distance covered between 7.00 and 10.99 km/h; *Z3*, distance covered between 11.00 and 14.99 km/h; *Z4*, distance covered between 15.00 and 18.99 km/h; *Z5*, distance covered > 19.00 km/h; ^*^p < 0.05; ^#^moderate correlation (r = 0.3–0.5)

## Discussion

This study aimed to (i) analyze the impact of a small-sided game training program in the locomotor profile of youth male soccer players (while interacting with the baseline level – higher and lower level); and (ii) test the relationships between variation in locomotor profile and the accumulated demands in 3v3, 5v5 and match over the period of observation. The main results showed that the baseline vs. post-observation comparison significantly differed significantly on VIFT and PSS, while no significant differences were found in ASR and TSA scores. However, when comparing players with lower TSA vs. higher TSA, no significant differences were found in VIFT, PSS, ASR, and TSA scores. Through the same design, no studies were found to characterize soccer players. Still, there are some studies that analyzed selected versus non-selected players and found that, in general, chosen players presented a better performance in sprint tests [45, 46]. Previous studies suggested that sprint is a determinant ability to select under-17 soccer players [45, 46]. These contrasting results could be explained by the fact that a TSA was used to categorize players in higher/lower scores which consequently may have influenced the results.

Moreover, significant correlations were found between PSS and match total distance, match Z2, and match Z3. Significant correlations were found between TSA and match total distance, match Z1, match Z2, and match Z3. Match demands play an exciting role in the association with positive adaptations in PSS and TSA (this last one is caused by PSS), which was not revealed in match Z4 and Z5, neither by 3v3 and 5v5 formats. Indeed, matches had been reported as the most demanding sessions of the week [47–49] and for that reason, it would be expected that match Z4 and Z5 would also correlate with PSS and TSA since they are associated with high-intensity action that is decisive to the results of the match [15].

Another justification for the lack of correlations between Z4 and Z5 with PSS could be associated with the thresholds for both zones (Z4: 15.00–18.99 km/h; Z5: >19.00 km/h). For example, a previous study used Z4 as 17.0–21 km/h and Z5 as > 21 km/h, and the authors could not find any correlations with changes in maximal aerobic speed and maximal sprint speed [27]. Even so, this contrasts with data from professional players where moderate relationships were found between VIFT with total distance, high-speed running (> 19.8 km/h), and sprint distance (≥ 25.2 km/h) [33].

The present study also failed to show significant associations between HR measures with physical fitness measures, which is also supported by previous research [50], but it is in contrast to a recent study by [33] that found a correlation between HR measures and changes in VIFT. The methodological differences between the studies can justify the different results. Nonetheless, our findings seem not to be influenced by HR measures.

VIFT and PSS improved over the three weeks. However, the such scenario was not so evident in the ASR and TSA (see Fig. 2). Possibly, the variations occurred in different players (for example, some improved PSS, while different ones improved VIFT) which may justify different findings in VIFT. Considering that both VIFT and PSS were a part of the calculation of ASR, it seems logical that there needed more time to improve ASR. Previous studies support the association between players with better PSS and better ASR [16, 51]. A longer study period would also provide better ASR, and/or the use of different tests could give different results in ASR, which should be recommended in future research.

Furthermore, there was not a dose-response relationship between load imposed in SSGs (3v3 and 5v5) and changes in the VIFT or PSS. Still, full match demands were found to be significantly correlated with variations in PSS. Despite the short duration of the intervention, there seems to be a close relationship between dose and changes. For instance, VIFT improved between 6.3 and 7.3% for both lower and high TSA players. These findings were corroborated by some studies despite the use of different measures and study durations [8, 26, 52]. For instance, the study of Clemente et al. [26] found an improvement of 7.5% in VO2max over six weeks. It justified it with continuous training in moderate-to-vigorous activities closely related to improving aerobic capacity.

Although our study did not consider all the training demands, a question that arises is related to the high/low responders and/or non-responders [30]. The analysis of the present study was based on means, which is common in research. Still, there are several inter variations between individuals that can influence results on variables such as maximal oxygen uptake [53], exercise HR [54], aerobic threshold [55], the anaerobic threshold [54, 55]. Thus, our results may be in similar conditions, which recommends further research to confirm the present results.

The present study presents some limitations that should be addressed: (a) the small sample size; (b) the brief period of time of observation; (c) TSA calculation was based only on three measures, VIFT, PSS, and ASR; (d) apart from matched, only 3v3 and 5v5 were considered for analysis; (e) only a short period of three weeks of the pre-season was analyzed. Since the sample is small and highly associated with a specific context, it is not recommended to perform generalizations of these findings. Bias related to the number of variables collected should also be highlighted. An enormous myriad of factors should be considered for a solid interpretation of the findings.

Future studies should develop their research with larger sample sizes and longer interventions. Moreover, TSA can be calculated with more fitness variables and other technical/tactical variables (i.e., shooting, passing, dribbling, ball control, and tactical skills) [45, 46]. In addition, complementary training, such as strength and conditioning sessions, should also be included for future analysis. As a practical implication, this study reveals that although the locomotor profile was improved after the introduction of small-sided game drills, it seems that the causes of the changes may not be exclusively related to the load imposed in these games. This suggests that coaches should consider that a diversity of factors influences fitness changes, and using small-sided games is only one more resource to introduce in the training plan.

## Conclusion

Considering that TSA calculation was based only on VIFT, PSS and ASR, the study’s first aim showed no differences between players with lower or higher TSA, which makes speculate that the variables used may not be enough to distinguish the athleticism of players. Even so, three weeks of intervention improved VIFT and PSS.

Regarding the second aim of the study, only matches showed associations between locomotor demands with TSA and PSS. However, high intensity running (> 15 km/h) locomotor did not show such associations, and thus it seems that high intensityhigh-intensity locomotor demands are not determinants to modulate changes over time.

## Supplementary Information

**Additional file 1**. Dataset of heart rate and locomotor responses during the 3v3 format

**Additional file 2**. Dataset of heart rate and locomotor responses during the 5v5 format

**Additional file 3**. Dataset of physical fitness assessment performed in the players

## Data Availability

All data generated or analyzed during this study are availbe as a supplemntry files.
